# Using Robinson-Foulds supertrees in divide-and-conquer phylogeny estimation

**DOI:** 10.1186/s13015-021-00189-2

**Published:** 2021-06-28

**Authors:** Xilin Yu, Thien Le, Sarah A. Christensen, Erin K. Molloy, Tandy Warnow

**Affiliations:** 1 Amazon AWS, Seattle, USA; 2 Department of EECS, Massachusetts Institute of Technology, Cambridge, USA; 3 Computer Science Department, University of Illinois at Urbana-Champaign, Urbana, USA

**Keywords:** Supertrees, Divide-and-conquer, Phylogeny estimation

## Abstract

One of the Grand Challenges in Science is the construction of the *Tree of Life*, an evolutionary tree containing several million species, spanning all life on earth. However, the construction of the Tree of Life is enormously computationally challenging, as all the current most accurate methods are either heuristics for **NP**-hard optimization problems or Bayesian MCMC methods that sample from tree space. One of the most promising approaches for improving scalability and accuracy for phylogeny estimation uses divide-and-conquer: a set of species is divided into overlapping subsets, trees are constructed on the subsets, and then merged together using a “supertree method”. Here, we present Exact-RFS-2, the first polynomial-time algorithm to find an optimal supertree of two trees, using the Robinson-Foulds Supertree (RFS) criterion (a major approach in supertree estimation that is related to maximum likelihood supertrees), and we prove that finding the RFS of three input trees is **NP**-hard. Exact-RFS-2 is available in open source form on Github at https://github.com/yuxilin51/GreedyRFS.

## Introduction

Supertree construction (i.e., the combination of a collection of trees, each on a potentially different subset of the species, into a tree on the full set of species) is a natural algorithmic problem that has important applications to computational biology; see [1] for a 2004 book on the subject and [2–9] for some of the recent papers on this subject. Supertree methods are particularly important for large-scale phylogeny estimation, where it can be used as a final step in a divide-and-conquer pipeline [10]: the species set is divided into two or more overlapping subsets, unrooted leaf-labelled trees are constructed (possibly recursively) on each subset, and then these subset trees are combined into a tree on the full dataset, using the selected supertree method. Furthermore, provided that optimal supertrees are computed, divide-and-conquer pipelines can be provably *statistically consistent* under stochastic models of evolution: i.e., as the amount of input data (e.g., sequence lengths when estimating gene trees, or number of gene trees when estimating species trees) increases, the probability that the true tree is returned converges to 1 [11, 12].

Unfortunately, the most accurate supertree methods are typically local-search heuristics for **NP**-hard optimization problems [2, 3, 7, 13–17], and are computationally intensive on large datasets. However, divide-and-conquer strategies, especially recursive ones, may only need to apply supertree methods to two trees at a time, and hence the computational complexity of supertree estimation given two trees is of interest. One optimization problem where optimal supertrees can be found on two trees is the **NP**-hard Maximum Agreement Supertree (SMAST) problem (also known as the Agreement Supertree Taxon Removal problem), which removes a minimum number of leaves so that the reduced trees have an agreement supertree [4, 6]. Similarly, the Maximum Compatible Supertree (SMCT) problem, which removes a minimum number of leaves so that the reduced trees have a compatibility supertree [18, 19], can also be solved in polynomial time on two trees (and note that SMAST and SMCT are identical when the input trees are fully resolved). Because SMAST and SMCT remove taxa, methods for these optimization problems are not *true supertree methods*, because they do not return a tree on the entire set of taxa. However, solutions to SMAST and SMCT could potentially be used as *constraints* for other supertree methods, where the deleted leaves are added into the computed SMAST or SMCT trees, so as to optimize the desired criterion.

When restricting to methods that return trees on the full set of taxa, much less seems to be understood about finding supertrees on two trees. However, if the two input trees are compatible (i.e., there is a supertree that equals or refines each tree when restricted to the respective leaf set), then finding that compatibility supertree is solvable in polynomial time, using (for example) the well-known BUILD algorithm [20], but more efficient algorithms exist (e.g., [19, 21]).

Since compatibility is a strong requirement (rarely seen in biological datasets), optimization problems are more relevant. One optimization problem worth discussing is the Maximum Agreement Supertree Edge Contraction problem (which takes as input a set of rooted trees and seeks a minimum number of edges to collapse so that an agreement supertree exists). This problem is **NP**-hard, but the decision problem can be solved in $O((2k)^p k n^2)$ time when the input has *k* trees and *p* is the allowed number of number of edges to be collapsed [4]. Note that the algorithm for MAST-EC proposed by [4] may be exponential even for two trees, when the number of edges that must be collapsed is $\Omega (n)$ (e.g., imagine two caterpillar trees, where one is obtained from the other by moving the left-most leaf to the rightmost position).

In sum, while supertree methods are important and well studied, when restricted to the major optimization problems that do not remove taxa, polynomial time methods do not seem to be available, even for the special case where the input contains just two trees. This restriction has consequences for large-scale phylogeny estimation, as without good supertree methods, divide-and-conquer pipelines are not guaranteed to be statistically consistent, are not fast, and do not have good scalability [12].

In this paper we examine the well-known Robinson-Foulds Supertree (RFS) problem [22], which seeks a supertree that minimizes the total Robinson-Foulds [23] distance to the input trees. Although RFS is **NP**-hard [24], it has several desirable properties: it is closely related to maximum likelihood supertrees [25] and, as shown very recently, has good theoretical performance for species tree estimation in the presence of gene duplication and loss [26]. Because of its importance, there are several methods for RFS supertrees, including PluMiST [5], MulRF [27], and FastRFS [28]. A comparison between FastRFS and other supertree methods (MRL [2], ASTRAL, ASTRID [29], PluMiST, and MulRF) on simulated and biological supertree datasets showed that FastRFS matched or improved on the other methods with respect to topological accuracy and RFS criterion scores [28]. Hence, FastRFS is currently the leading method for the RFS optimization problem.

The main contributions of this paper are:

- We present Exact-2-RFS, a polynomial time algorithm for the Robinson-Foulds Supertree (RFS) of two trees, which establishes that RFS is solvable in $O(n^2 |X|)$ time for two trees, where *n* is the number of leaves and *X* is the set of shared leaves (Theorem 1). We also show that RFS is **NP**-hard for three or more trees (Lemma 5).
- We prove that divide-and-conquer pipelines using Exact-RFS-2 are statistically consistent methods for phylogenetic tree estimation (both gene trees and species trees) under standard evolutionary models (Theorem 2).
- We establish the relationships between RFS and other supertree problems (Sect. 2.1), showing that it is equivalent to some other problems but not to all.

The remainder of the paper is organized as follows. In Sect. 1, we provide terminology and define the optimization problems we consider. We present the Exact-RFS-2 algorithm and establish theory related to the algorithm in Sect. 2. We conclude in Sect. 3 with a summary of our results and a discussion regarding future research directions.

## Terminology and problem statements

We let $[N]=\{1,2,\ldots ,N\}$ and ${\mathcal {A}}=\{T_i\mid i \in [N]\}$ denote the input to a supertree problem, where each $T_i$ is a phylogenetic tree on leaf set $L(T_i)=S_i \subseteq S$ (where *L*(*t*) denotes the leaf set of *t*) and the output is a tree *T* where *L*(*T*) is the set of all species that appear as a leaf in at least one tree in ${\mathcal {A}}$, which we will assume is all of *S*. We use the standard supertree terminology, and refer to the trees in ${\mathcal {A}}$ as “source trees” and the set ${\mathcal {A}}$ as a “profile”. For a tree *T*, let *V*(*T*) and *E*(*T*) denote the set of vertices and edges of *T*, respectively.

*Robinson-Foulds Supertree* Each edge *e* in a tree *T* defines a bipartition $\pi _e := [A|B]$ of the leaf set, and each tree is defined by the set $C(T) := \{\pi _e \mid e\in E(T)\}$. The *Robinson-Foulds distance* [23] (also called the bipartition distance) between trees *T* and $T'$ with the same leaf set is $\text {RF}(T, T') := |C(T)\backslash C(T')| + |C(T') \backslash C(T)|$. We extend the definition of RF distance to allow for *T* and $T'$ to have different leaf sets as follows: $RF(T,T'):=RF(T|_X, T'|_X)$, where *X* is the shared leaf set and $t|_X$ denotes the homeomorphic subtree of *t* induced by *X*. Letting ${\mathcal {T}}_S$ denote the set of all phylogenetic trees such that $L(T) = S$ and ${\mathcal {T}}^B_S$ denote the binary trees in ${\mathcal {T}}_S$, then a Robinson-Foulds supertree [22] of a profile ${\mathcal {A}}$ is a binary tree

$$\begin{aligned} T_{\textsc {RFS}} = \mathop {\mathrm{argmin}}\limits _{T \in {\mathcal {T}}^B_S} \sum _{i \in [N]} \text {RF}(T, T_i). \end{aligned}$$

We let $\text {RF}(T,{\mathcal {A}}):=\sum _{i \in [N]} \text {RF}(T, T_i)$ denote the *RFS score* of *T* with respect to profile ${\mathcal {A}}$. Thus, the **Robinson-Foulds Supertree problem** takes as input the profile ${\mathcal {A}}$ and seeks a Robinson-Foulds (RF) supertree for ${\mathcal {A}}$, which we denote by RFS$({\mathcal {A}})$.

*Split Fit Supertree* The Split Fit (SF) Supertree problem was introduced in [30], and is based on optimizing the number of shared splits (i.e., bipartitions) between the supertree and the source trees. For two trees *T*, $T'$ with the same leaf set, the *split support* is the number of shared bipartitions, i.e., $\text {SF}(T, T') := |C(T) \cap C(T')|$. For trees with different leaf sets, we restrict them to the shared leaf set before calculating the split support. The Split Fit supertree for a profile ${\mathcal {A}}$ of source trees, denoted $\textsc {SFS}$(${\mathcal {A}}$), is a tree $T_{\textsc {SFS}} \in {\mathcal {T}}^B_S$ such that

$$\begin{aligned} T_{\textsc {SFS}} = \mathop {\mathrm{argmax}}\limits _{T \in {\mathcal {T}}^B_S} \sum _{i \in [N]} \text {SF}(T, T_i). \end{aligned}$$

Thus, the split support score of *T* with respect to ${\mathcal {A}}$ is $\text {SF}(T,{\mathcal {A}}):=\sum _{i\in [N]}\text {SF}(T, T_i)$. The **Split Fit Supertree (SFS) problem** takes as input the profile ${\mathcal {A}}$ and seeks a Split Fit supertree (the supertree with the maximum split support score), which we denote by $\textsc {SFS}$(${\mathcal {A}}$).

*Nomenclature for variants of* $\textsc {RFS}$ *and* $\textsc {SFS}$ *problems*

- The relaxed versions of the problems where we do not require the output to be binary (i.e., we allow $T \in {\mathcal {T}}_S$) are named $\textsc {Relax}$—$\textsc {RFS}$ and $\textsc {Relax}$—$\textsc {SFS}$.
- We append “-*N*” to the name to indicate that we assume there are *N* source trees. If no number is specified then the number of source trees is unconstrained.
- We append “-B” to the name to indicate that the source trees are required to be binary; hence, we indicate that the source trees are allowed to be non-binary by not appending -$\textsc {B}$.

Thus, the RFS problem with two binary input trees is $\textsc {RFS-2}$-$\textsc {B}$ and the relaxed SFS problem with three (not necessarily binary) input trees is $\textsc {Relax}$—$\textsc {SFS-3}$.

*Other notation* For any $v\in V(T)$, we let $N_T(v)$ denote the set of neighbors of *v* in *T*. A tree $T'$ is a *refinement* of *T* iff *T* can be obtained from $T'$ by contracting a set of edges. Two bipartitions $\pi _1$ and $\pi _2$ of the same leaf set are said to be *compatible* if and only if there exists a tree *T* such that $\pi _i \in C(T), i=1,2$. A bipartition $\pi = [A|B]$ restricted to a subset *R* is $\pi |_R = [A\cap R | B\cap R]$. For a graph *G* and a set *F* of vertices or edges, we use $G + F$ to represent the graph obtained from adding the set *F* of vertices or edges to *G*, and $G-F$ is defined for deletions, similarly.

## Theoretical results

In this section we establish the main theoretical results, including the relationship between supertree problems (Sect. 2.1), the proof that RFS for 3 trees is NP-hard (Sect. 2.2), the polynomial time Exact-2-RFS algorithm (Sect. 2.3), and the use of this algorithm within divide-and-conquer pipelines for statistically consistent phylogeny estimation (Sect. 2.4).

### Relationships between supertree problems

This section establishes the relationships between the different supertree problems. We establish that some supertree problems have the same optimal solutions, others do not, etc. We begin by establishing the equivalence between the $\textsc {RFS}$and $\textsc {SFS}$supertree problems.

#### **Lemma 1**

*Given an input set* ${\mathcal {A}}$* of source trees, a tree* $T \in {\mathcal {T}}^B_S$* is an optimal solution for* $\textsc {RFS}$(${\mathcal {A}}$)* if and only if it is an optimal solution for* $\textsc {SFS}$(${\mathcal {A}})$.

#### *Proof*

Let $T_1,T_2,\dots ,T_N$ and $S_1,S_2,\dots ,S_N$ be defined as from problem statement of $\textsc {RFS}$. Let *T* be any binary tree of leaf set *S*. Then $T|_{S_i}$ is also binary and thus $|C(T|_{S_i})| = 2|S_i| -3$. For any $i \in [N]$, we have

$$\begin{aligned}&\text {RF}(T,T_i)+2\text {SF}(T,T_i) \\ =&|C(T|_{S_i})\backslash C(T_i)| + |C(T_i) \backslash C(T|_{S_i})| + 2|C(T|_{S_i}) \cap C(T_i)| \\ =&|C(T|_{S_i})\backslash C(T_i) \cup (C(T|_{S_i}) \cap C(T_i))| + |C(T_i) \backslash C(T|_{S_i}) \cup (C(T|_{S_i}) \cap C(T_i))|\\ =&|C(T|_{S_i})| + |C(T_i)|\\ =&2|S_i| -3 + |C(T_i)|. \end{aligned}$$

Taking the sum of the equations over $i \in [N]$, we have

$$\begin{aligned}&\sum _{i \in [N]} [\text {RF}(T|_{S_i}, T_i) + 2\text {SF}(T|_{S_i},T_i)] = \sum _{i \in [N]} ( 2|S_i| -3 + |C(T_i)|), \end{aligned}$$

which is a constant (i.e., it does not depend on the tree *T*).

Therefore, for any binary tree *T* and any profile ${\mathcal {A}}$ of source trees, the sum of *T*’s RFS score and twice *T*’s split support score is the same, independent of *T*. This implies that minimizing the RFS score is the same as maximizing the split support score. Although this argument depends on the output tree being binary, it does not depend on the input trees being binary. Hence, we conclude that $\textsc {RFS}$ and $\textsc {SFS}$ have the same set of optimal supertrees. $\square$

In contrast with Lemma 1, we will show that $\textsc {Relax}$—$\textsc {RFS}$ and $\textsc {Relax}$—$\textsc {SFS}$ are not equivalent.

#### **Lemma 2**

*There exist instances of* $\textsc {Relax}$—$\textsc {RFS}$* and* $\textsc {Relax}$—$\textsc {SFS}$* in which an optimal solution to* $\textsc {Relax}$—$\textsc {RFS}$* is not an optimal solution to* $\textsc {Relax}$—$\textsc {SFS}$*, and vice-versa.*

#### *Proof*

Let $N \ge 5$ be any integer. Let $\pi _i =[1,2,\dots ,i+1 \mid i+2,\dots ,N]$ for any $i \in [N-3]$. Let ${\mathcal {A}} = \{T_1, T_2, \dots , T_{n-3}\}$ be a profile, where for all $i \in [N-3]$, the leaf set of $T_i$ is [*N*] and $T_i$ contains a single internal edge defining $\pi _i$. Let $\Pi _{[N]}$ denote the set of trivial bipartitions of [*N*]. Let *T* be the star tree with leaf set [*N*] (i.e., *T* has no internal edges). We note that $C(T) = \Pi _{[N]}$. Let $\Pi '= \{\pi _i\mid i \in [N-3]\}$ (i.e., $\Pi '$ contains all the nontrivial bipartitions from the trees in ${\mathcal {A}}$). Let $T'$ be the caterpillar tree on leaf set [*N*] (i.e., $T'$ is formed by taking a path of length $N-2$ with vertices $v_2, v_3, \ldots , v_{N-1}$ in that order, and making leaf 1 adjacent to $v_2$, leaf *i* adjacent to $v_i$, and leaf *N* adjacent to $v_{N-1}$). We note that $T'$ is the unique tree such that $C(T') = \Pi _{[N]} \cup \Pi '$ and thus a compatibility supertree for ${\mathcal {A}}$.

We will show that (1) *T* is an optimal solution for $\textsc {Relax}$—$\textsc {RFS}$(${\mathcal {A}}$), but not an optimal solution for $\textsc {Relax}$—$\textsc {SFS}$(${\mathcal {A}}$), and (2) that $T'$ is an optimal solution for $\textsc {Relax}$—$\textsc {SFS}$(${\mathcal {A}}$), but not an optimal solution for $\textsc {Relax}$—$\textsc {RFS}$(${\mathcal {A}}$). (1) We first show that *T* is not an optimal solution for $\textsc {Relax}$—$\textsc {SFS}$(${\mathcal {A}}$). Since *T*‘ is a compatibility supertree of trees in ${\mathcal {A}}$, it achieves the maximum split support score possible. In particular, $C(T') \cap C(T_i) = \Pi _{[N]} \cup \{\pi _i\}$ and thus $\text {SF}(T',T_i) = N+1$ for all $i \in [N-3]$. Overall, the split support score of $T'$ is

1 $$\begin{aligned} \text {SF}(T',{\mathcal {A}}) = \sum _{i \in [N-3]}\text {SF}(T',T_i) = (N-3)(N+1).\end{aligned}$$

Since $C(T) \cap C(T_i) = \Pi _{[N]}$, we have

2 $$\begin{aligned} \text {SF}(T,{\mathcal {A}}) = \sum _{i \in [N-3]}\text {SF}(T,T_i) = (N-3)N < (N-3)(N+1)\end{aligned}$$

for any $N \ge 5$. Therefore, *T* is not an optimal solution for $\textsc {Relax}$—$\textsc {SFS}$(${\mathcal {A}}$).

Next, we show that *T* is an optimal solution to $\textsc {Relax}$—$\textsc {RFS}$(${\mathcal {A}}$). Since $|C(T) \backslash C(T_i)| + |C(T_i) \backslash C(T)| = 1$ for all $i \in [N-3]$, the RFS score of *T* is

3 $$\begin{aligned} \text {RF}(T,{\mathcal {A}}) = \sum _{i \in [N-3]}\text {RF}(T,T_i) = N-3. \end{aligned}$$

Now consider any tree $t \ne T$ with leaf set [*N*], and suppose *t* contains *p* bipartitions in $\Pi '$ and *q* bipartitions in $2^{[N]} \backslash (\Pi ' \cup \Pi _{[N]})$ where $p, q \in {\mathbb {N}}$. Since $t \ne T$, at least one of *p* and *q* is nonzero. Therefore,

4 $$\begin{aligned} \text {RF}(t,{\mathcal {A}}) =&\sum _{i \in [N-3]} \text {RF}(t, T_i) \end{aligned}$$

5 $$\begin{aligned} =&\sum _{i \in [N-3]} |C(t)\backslash C(T_i)| + |C(T_i)\backslash C(t)| \end{aligned}$$

6 $$\begin{aligned} =&q(N-3)+(p-1)p + p(N-3-p) + (N-3-p) \end{aligned}$$

7 $$\begin{aligned} =&(N-3)+q(N-3) + p(N-5). \end{aligned}$$

Since $N \ge 5$ and both *p* and *q* are non-negative with at least one of them nonzero, we know the RFS score of *t* is strictly greater than that of *T*. Therefore, *T* is an optimal solution to $\textsc {Relax}$—$\textsc {RFS}$(${\mathcal {A}}$).

For (2), the analysis above already shows that $T'$ has the largest possible split support score. Hence, $T'$ is an optimal solution to the relaxed Split Fit Supertree problem. However, the RFS score for the star tree *T* is $N-3$ and the RFS score for $T'$ is $(N-4)(N-3)$, which is strictly larger than $N-3$ for $N>5$; hence, $T'$ is not an optimal solution for the relaxed RF supertree problem. $\square$

We show that the Split Fit Supertree problem and the Asymmetric Median Supertree ($\textsc {AMS}$) problem, which was introduced in [31] and which we will present below, have the same set of optimal solutions and thus the hardness of one implies hardness of another.

The Asymmetric Median Supertree problem takes a profile ${\mathcal {A}}=\{T_1,T_2,\dots ,T_N\}$ with leaf sets $S_i$ for $T_i$ and finds a binary tree $T^*$ on leaf set $S := \bigcup _{i \in [N]} S_i$ such that

8 $$\begin{aligned} T^* = \mathop {\mathrm{argmin}}\limits _{T \in {\mathcal {T}}_S} \sum _{i \in [N]} |C(T_i) \setminus C(T|_{S_i})|. \end{aligned}$$

In other words, the asymmetric median supertree $T^*$ minimizes the total number of bipartitions that are in the source trees and not in the supertree (equivalently, it minimizes the total number of false negatives).

#### **Lemma 3**

*Given a profile* ${\mathcal {A}}=\{T_1,T_2,\dots ,T_N\}$ *of source trees with leaf sets* $S_i$ *for* $T_i$ *and* $S := \bigcup _{i \in [N]} S_i$* , a tree* $T \in {\mathcal {T}}_S$ *is a Split Fit Supertree for* ${\mathcal {A}}$ *if and only if it is an Asymmetric Median Supertree for* ${\mathcal {A}}$.

#### *Proof*

Let $\textsc {FN} (T, {\mathcal {A}}) = \sum _{i \in [N]} |C(T_i) \setminus C(T|_{S_i})|$ be the total number of false negatives of *T* with respect to ${\mathcal {A}}$, and we refer to this as the false negative score of *T*. Then,

9 $$\begin{aligned} \text {SF}(T,{\mathcal {A}}) + \textsc {FN} (T, {\mathcal {A}}) =&\sum _{i \in [N]}| C(T_i) \cap C(T|_{S_i})| + |C(T_i) \backslash C(T|_{S_i})| \end{aligned}$$

10 $$\begin{aligned} =&\sum _{i \in [N]} |C(T_i)|. \end{aligned}$$

Since the sum of the split support score of *T* and the false negative score of *T* is the same, regardless of *T*, minimizing the false negative score is the same as maximizing the split support score. Hence any tree *T* is an Asymmetric Median supertree if and only if it is a Split Fit supertree, for all profiles ${\mathcal {A}}$. $\square$

Recall that the SMAST and SMCT problems seek trees that are obtained after deleting minimal numbers of leaves from the input trees so that an agreement supertree or compatible supertree can be constructed from the reduced input trees. Here, we examine the possibility of using these output trees as constraint trees on the search for RFS supertrees, so that the removed taxa could be introduced into the constraint trees. We show that exact solutions to the SMAST and SMCT (Maximum Agreement Supertree and Maximum Compatible Supertree) problems are not directly relevant to solving the Robinson-Foulds supertree problem.

#### **Lemma 4**

*There exists a pair of binary trees* $T_1$ *and* $T_2$ *for which some optimal SMAST or SMCT supertree cannot be extended to any optimal RFS supertree through the insertion of missing taxa.*

#### *Proof*

Let $T_1$ and $T_2$ be unrooted trees, with $T_1$ given by the Newick string (*A*, ((*B*, *x*), ((*C*, *y*), (*D*, *E*)))) and $T_2$ given by (*A*, (*C*, (*z*, (*B*, (*D*, *E*))))). An RFS supertree for this pair $(T_1,T_2)$ is given by (*A*, ((*C*, *y*), (*z*, ((*B*, *x*), (*D*, *E*))))),  and has total RF distance to $T_1$ and $T_2$ equal to 2. Note that at least one of A,B,C must be deleted to form an agreement supertree. Suppose C is deleted. Then ((*A*, *z*), ((*B*, *x*), (*y*, (*D*, *E*)))) is an optimal SMAST. Observe that any way of adding C into this tree produces a supertree that has total RFS score greater than 2. Hence, for this pair of input trees, for at least one optimal SMAST supertree, there is no way to extend that optimal supertree into an optimal RFS supertree. The same proof follows for the SMCT problem, since SMCT and SMAST are identical when the input trees are fully resolved (binary). $\square$

### NP-hardness results

We establish that some supertree problems are NP-hard.

#### **Lemma 5**

$\textsc {RFS-3}$, $\textsc {SFS-3}$*, and* $\textsc {Relax}$—$\textsc {SFS-3}$* are all* ***NP****-hard.*

#### *Proof*

By Lemma 3 and Lemma 1, we know that for any profile ${\mathcal {A}}$, the Robinson-Foulds, Split Fit, and Asymmetric Median supertrees all have the same set of optimal solutions. We also note that the Asymmetric Median Tree problem was shown to be **NP**-hard for three trees [32], which is the same as the Asymmetric Median Supertree problem when all three trees have the same set of species. Therefore, $\textsc {SFS-3}$ and $\textsc {RFS-3}$ are both **NP**-hard. Since refining a tree never decreases its split support score, $\textsc {SFS-3}$ trivially reduces to $\textsc {Relax}$—$\textsc {SFS-3}$, and thus $\textsc {Relax}$—$\textsc {SFS-3}$ is also **NP**-hard. $\square$

### Solving RFS and SFS on two binary trees

The main result of this paper is Theorem 1 and the polynomial time algorithm, Exact-RFS-2, for $\textsc {RFS}$ and $\textsc {SFS}$ of two binary trees.

#### **Theorem 1**

*Let* ${\mathcal {A}} = \{T_1,T_2\}$ *with* $S_i$ *the leaf set of* $T_i$ ($i=1,2$*) and* $X:= S_1 \cap S_2$*. The problems* $\textsc {RFS-2}$-$\textsc {B}$(${\mathcal {A}}$*) and* $\textsc {SFS-2}$-$\textsc {B}$(${\mathcal {A}}$*) can be solved in* $O(n^2|X|)$ *time, where* $n :=\max \{|S_1|,|S_2|\}$.

The proof for Theorem 1 is provided later; here we present the algorithm, Exact-RFS-2, which we use to establish Theorem 1.

The input to Exact-RFS-2 is a pair of binary trees $T_1$ and $T_2$. Let *X* denote the set of shared leaves. At a high level, Exact-RFS-2 constructs a tree $T_{\text {init}}$ that has a central node that is adjacent to every leaf in *X* and to the root of every “rooted extra subtree” (a term we define below under “Additional notation”) so that $T_{\text {init}}$ contains all the leaves in *S*. It then modifies $T_{\text {init}}$ by repeatedly refining it to add specific desired bipartitions, to produce an optimal Split Fit (and optimal Robinson-Foulds) supertree (Fig. 3). The bipartitions that are added are defined by a maximum independent set in a bipartite “weighted incompatibility graph” we compute.

**Fig. 1:**
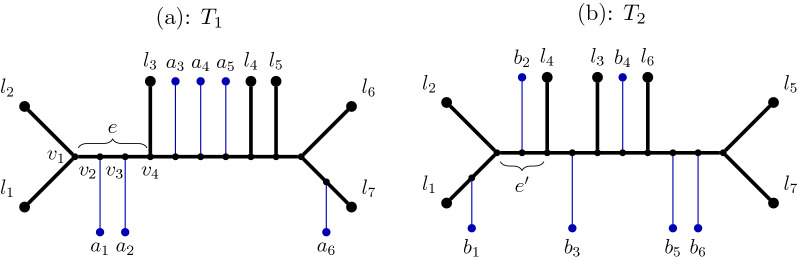
$T_1$ and $T_2$ (depicted in (**a**) and (**b**), respectively) have an overlapping leaf set $X = \{l_1,l_2,\dots ,l_7\}$. Each of $a_1,\dots , a_6$ and $b_1,\dots , b_6$ can represent a multi-leaf extra subtree. For $e \in T_1|_X$ as shown, *P*(*e*) is the path from $v_1$ to $v_4$, so $w(e) = 3$. Using indices to represent the shared leaves, let $\pi = [12 | 34567]$; then $e_1(\pi ) = e$ and $e_2(\pi ) = e'$. $\mathcal {TR}(e) = \{a_1,a_2\}$, $\mathcal {TR}(e') = \{b_2\}$, so $\mathcal {TR}^*(\pi ) = \{a_1,a_2,b_2\}$. Let $A = \{1,2,3\}$, $B = \{4,5,6,7\}$. Ignoring the trivial bipartitions, we have $\mathcal {BP}(A) = \{[12|34567] \}$ and $\mathcal {BP}(B) = \{[1234|567],[12345|67],[12346|57]\}$. $\mathcal {TRS}(A) = \{a_1,a_2,b_1,b_2\}$ and $\mathcal {TRS}(B) = \{ a_6,b_4,b_5,b_6\}$

**Fig. 2:**
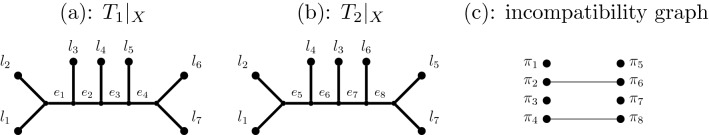
We show (**a**) $T_1|_X$, (**b**) $T_2|_X$, and (**c**) their incompatibility graph, based on the trees $T_1$ and $T_2$ in Fig. 1 (without the trivial bipartitions). Each $\pi _i$ is the bipartition induced by $e_i$, and the weights for $\pi _1,\dots , \pi _8$ are 3, 4, 1, 1, 2, 2, 2, 3, in that order. We note that $\pi _1$ and $\pi _5$ are the same bipartition, but they have different weights as they are induced by different edges; similarly for $\pi _3$ and $\pi _7$. The maximum weight independent set in this graph has all the isolated vertices ($\pi _1, \pi _3, \pi _5, \pi _7$) and also $\pi _2,\pi _8$, and so has total weight 15

**Fig. 3:**
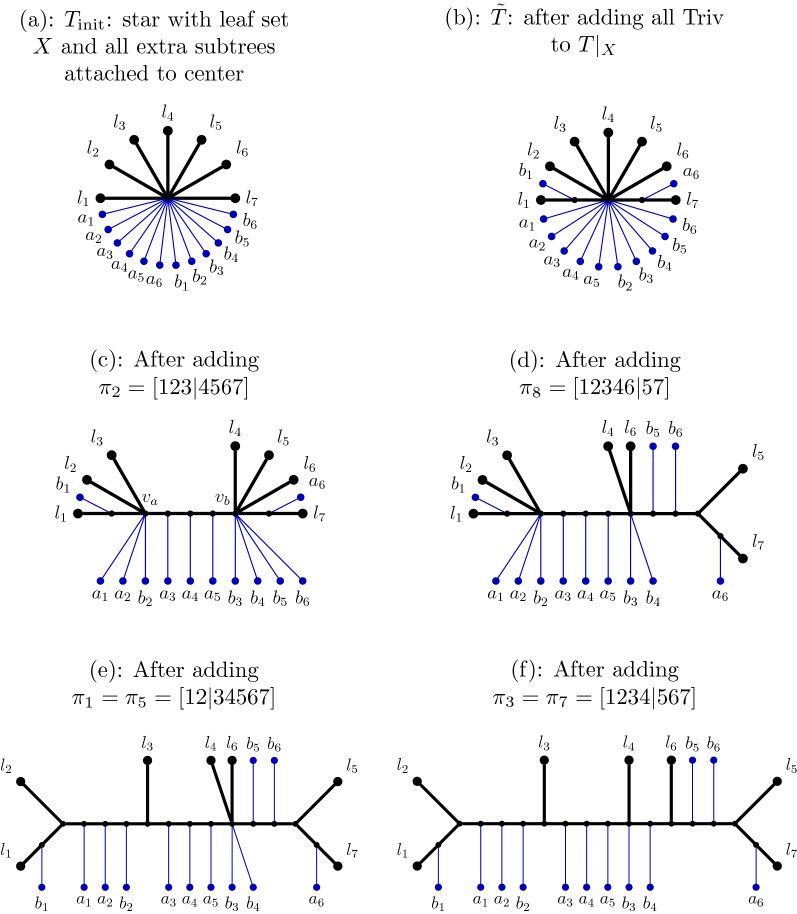
Algorithm 1 working on $T_1$ and $T_2$ from Fig. 1 as source trees; the indices of leaves in $X =\{l_1,l_2,\dots ,l_7\}$ represent the leaves and the notation of $\pi _1,\dots ,\pi _8$ is from Fig. 2. In (**a**)–(**f**), the $p_1(\cdot )$ score of the trees are 14, 16, 20, 23, 27, 29, in that order. We explain how the algorithm obtains the tree in **c** from ${\tilde{T}}$ by adding $\pi _2 = [123|4567]$ to the backbone of ${\tilde{T}}$. Let $A =\{l_1,l_2,l_3\}$ and $B = \{l_4,l_5,l_6,l_7\}$. The center vertex *c* of ${\tilde{T}}$ is split into two vertices $v_a,v_b$ with an edge between them. Then all neighbors of *c* between *c* and *A* are made adjacent to $v_a$ while the neighbors between *c* and *B* are made adjacent to $v_b$. All neighbors of *c* which are roots of extra subtrees are moved around such that all extra subtrees in $\mathcal {TR}^*(\pi _2)$ are attached onto $(v_a,v_b)$; all extra subtrees in $\mathcal {TRS}(A) = \{a_1, a_2, b_2\}$ are attached to $v_a$ and all extra subtrees in $\mathcal {TRS}(B) = \{b_4,b_5,b_6\}$ are attached to $v_b$. We note that in this step, $b_3$ can attach to either $v_a$ or $v_b$ because it is not in $\mathcal {TRS}(A)$ or $\mathcal {TRS}(B)$. However, when obtaining the tree in **d** from **c**, $b_3$ can only attach to the left side because for $A' = \{l_1,l_2,l_3,l_4,l_6\}$, $[124|3567] \in \mathcal {BP}(A')$ and thus $b_3 \in \mathcal {TRS}(A')$

*Additional notation* Let $2^X$ denote the set of all bipartitions of *X*; any bipartition that splits a single leaf from the remaining $|X|-1$ leaves will be called “trivial” and the others will be called “non-trivial”. Let $C(T_1,T_2,X)$ denote $C(T_1|_X) \cup C(T_2|_X)$, and let $\text {Triv}$ and $\text {NonTriv}$ denote the sets of trivial and non-trivial bipartitions in $C(T_1, T_2, X)$, respectively. We refer to $T_i|_X, i=1,2$ as **backbone trees** (Fig. 2). Recall that we suppress degree-two vertices when restricting a tree $T_i$ to a subset *X* of the leaves; hence, every edge *e* in $T_i|_X$ will correspond to an edge or a path in *T* (see Fig. 1 for an example). We will let *P*(*e*) denote the path associated to edge $e \in T_i|_X$, and let $w(e):= |P(e)|$ (the number of edges in *P*(*e*)). Finally, for $\pi \in C(T_i|_X)$, we define $e_i(\pi )$ to be the edge that induces $\pi$ in $T_i|_X$ (Fig. 1).

The next concept we introduce is the set of **extra subtrees**, which are rooted subtrees of $T_1$ and $T_2$, formed by deleting *X* and all the edges and vertices on paths between vertices in *X* (i.e., we delete $T_i|_X$ from $T_i$). Each component in $T_i-T_i|_X$ is called an **extra subtree** of $T_i$, and the extra subtree *t* is naturally seen as rooted at the unique vertex *r*(*t*) that is adjacent to a vertex in $T_i|_X$. Thus, $\hbox {Extra}(T_i) = \{t \mid t \text { is a component in } T_i - T_i|_X\}.$ Note that if *X* contains the leaves of $T_i$ then there are no extra subtrees associated to $T_i$ for *X*.

We can now define the initial tree $T_{\text {init}}$ computed by Exact-RFS-2: $T_{\text {init}}$ has a center node that is adjacent to every $x \in X$ and also to the root *r*(*t*) for every extra subtree $t \in \hbox {Extra}(T_1) \cup \hbox {Extra}(T_2)$. Note that $T_{\text {init}}$ has a leaf for every element in *S*, and that $T_{\text {init}}|_{S_i}$ is a contraction of $T_i$, formed by collapsing all the edges in the backbone tree $T_i|_X$.

We say that an extra subtree *t* is **attached to edge** $e \in E(T_i|_X)$ if the root of *t* is adjacent to an internal node of *P*(*e*), and we let $\mathcal {TR}(e)$ denote the set of such extra subtrees attached to edge *e*. Similarly, if $\pi \in C(T_1,T_2,X)$, we let $\mathcal {TR}^*(\pi )$ refer to the set of extra subtrees that attach to edges in a backbone tree that induce $\pi$ in either $T_1|_X$ or $T_2|_X$. For example, if both trees $T_1$ and $T_2$ contribute extra subtrees to $\pi$, then $\mathcal {TR}^*(\pi ) := \bigcup _{i \in [2]} \mathcal {TR}(e_i(\pi )).$

For any $Q \subseteq X$, we let $\mathcal {BP}_i(Q)$ denote the set of bipartitions in $C(T_i|_X)$ that have one side being a strict subset of *Q*, and we let $\mathcal {TRS}_i(Q)$ denote the set of extra subtrees associated with these bipartitions. In other words, $\mathcal {BP}_i(Q) := \{ [A|B] \in C(T_i|_X) \mid A \subsetneq Q \text { or } B \subsetneq Q\}$, and $\mathcal {TRS}_i(Q) := \bigcup _{\pi \in \mathcal {BP}_i(Q)} \mathcal {TR}(e_i(\pi )).$ Intuitively, $\mathcal {TRS}_i(Q)$ denotes the set of extra subtrees in $T_i$ that are “on the side of *Q*”. Note that for any $\pi = [A|B] \in C(T_i|_X)$, $\mathcal {BP}_i(A) \cup \mathcal {BP}_i(B)$ is the set of bipartitions in $C(T_i|_X)$ that are compatible with $\pi$. Finally, let $\mathcal {BP}(Q) = \mathcal {BP}_1(Q)\cup \mathcal {BP}_2(Q), \text { and }\mathcal {TRS}(Q) = \mathcal {TRS}_1(Q) \cup \mathcal {TRS}_2(Q).$ We give an example for these terms in Fig. 1.

The *incompatibility graph* of a set of trees, each on the same set of leaves, has one vertex for each bipartition in any tree (and note that bipartitions can appear more than once) and edges between bipartitions if they are incompatible (see [32]). We compute a **weighted incompatibility graph** for the pair of trees $T_1|_X$ and $T_2|_X$, in which the weight of the vertex corresponding to bipartition $\pi$ appearing in tree $T_i|_X$ is $w(e_i(\pi ))$, as defined previously. Thus, if a bipartition is common to the two trees, it produces two vertices in the weighted incompatibility graph, and each vertex has its own weight (Fig. 2).

We divide ${\mathcal {C}}=C(T_1) \cup C(T_2)$ into two sets: $\Pi _1 = \{ [A|B] \in {\mathcal {C}} \mid A\cap X \ne \emptyset \text { and } B\cap X \ne \emptyset \}$, and $\Pi _2 = \{ [A|B] \in {\mathcal {C}} \mid A\cap X = \emptyset \text { or } B \cap X = \emptyset \}$. Intuitively, $\Pi _1$ is the set of bipartitions from the input trees that are induced by edges in the minimal subtree of $T_1$ or $T_2$ spanning *X*, and $\Pi _2$ are all the other input tree bipartitions. We define $p_1(\cdot )$ and $p_2(\cdot )$ on trees $T \in {\mathcal {T}}_S$ by:

$$\begin{aligned} p_1(T)&= \sum _{i \in [2]}|C(T|_{S_i}) \cap C(T_i) \cap \Pi _1|, \quad p_2(T) = \sum _{i \in [2]} |C(T|_{S_i}) \cap C(T_i) \cap \Pi _2|. \end{aligned}$$

Note that $p_1(T)$ and $p_2(T)$ decompose the split support score of *T* into the score contributed by bipartitions in $\Pi _1$ and the score contributed by bipartitions in $\Pi _2$; thus, the split support score of *T* with respect to $T_1,T_2$ is $p_1(T) + p_2(T)$.

As we will show, the two scores can be maximized independently and we can use this observation to refine $T_{\text {init}}$ so that it achieves the optimal total score.

*Overview of Exact-RFS-2 *
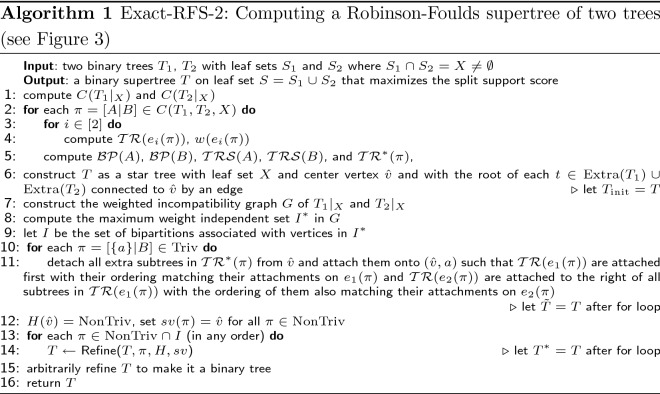


Exact-RFS-2 (Algorithm 1) has four phases. In the pre-processing phase (lines 1–5), it computes the weight function *w* and the mappings $$\mathcal {TR}, \mathcal {TR}^*, \mathcal {BP}$$, and $$\mathcal {TRS}$$ for use in latter parts of Algorithm 1 and Algorithm 2. In the initial construction phase (line 6), it constructs a tree $$T_{\text {init}}$$ (as described earlier), and we note that $$T_{\text {init}}$$ maximizes $$p_2(\cdot )$$ score (Lemma 7). In the refinement phase (lines 7–14), it refines $$T_{\text {init}}$$ so that it attains the maximum $$p_1(\cdot )$$ score, without changing the $$p_2(\cdot )$$ score. In the last phase (line 15), it arbitrarily refines *T* to make it binary. The refinement phase begins with the construction of a weighted incompatibility graph *G* of $$T_1|_X $$ and $$T_2|_X$$ (see Fig. 2). It then finds a maximum weight independent set of *G* that defines a set $$I \subseteq C(T_1, T_2, X)$$ of compatible bipartitions of *X*. Finally, it uses these bipartitions of *X* in *I* to refine $$T_{\text {init}}$$ to achieve the optimal $$p_1(\cdot )$$ score, by repeatedly applying Algorithm 2 for each $$\pi \in I$$ (and we note that the order does not matter). See Fig. 3 for an example of Exact-RFS-2 given two input source trees.

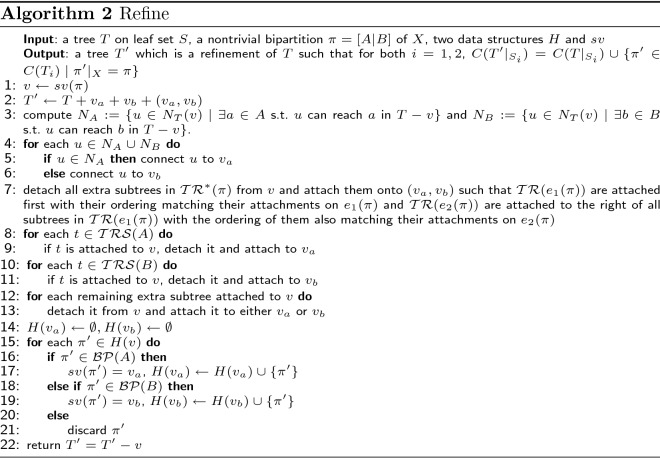



Algorithm 2 refines the given tree *T* on leaf set *S* with bipartitions on *X* from $C(T_1, T_2, X) \setminus C(T|_X)$. Given bipartition $\pi =[A|B]$ on *X*, Algorithm 2 produces a refinement $T'$ of *T* such that $C(T'|_{S_i}) = C(T|_{S_i}) \cup \{\pi ' \in C(T_i) \mid \pi '|_X= \pi \}$ for both $i=1,2$. To do this, we first find the unique vertex *v* such that no component of $T - v$ has leaves from both *A* and *B*. We create two new vertices $v_a$ and $v_b$ with an edge between them. We divide the neighbor set of *v* into three sets: $N_A$ is the set of neighbors that split *v* from leaves in *A*, $N_B$ is the set of neighbors that split *v* from leaves in *B*, and $N_{\text {other}}$ contains the remaining neighbors. Then, we make vertices in $N_A$ adjacent to $v_a$ and vertices in $N_B$ adjacent to $v_b$. We note that $N_{\text {other}} = \emptyset$ if $X = S$ and thus there are no extra subtrees. In the case where $X \ne S$, $N_{\text {other}}$ contains the roots of the extra subtrees adjacent to *v* and we handle them in four different cases to make $T'$ include the desired bipartitions:

- those vertices that root extra subtrees in $\mathcal {TR}^*(\pi )$ are moved onto the edge $(v_a,v_b)$ (by subdividing the edge to create new vertices, and then making these vertices adjacent to the new vertices)
- those vertices that root extra subtrees in $\mathcal {TRS}(A)$ are made adjacent to $v_a$
- those that root extra subtrees in $\mathcal {TRS}(B)$ are made adjacent to $v_b$
- the remaining vertices can be made adjacent to either $v_a$ or $v_b$

Algorithms 1 and 2 also use two data structures (functions) *H* and *sv*: (1) For a given node $v \in V(T)$, $H(v) \subseteq C(T_1,T_2,X)$ is the set of bipartitions of *X* that can be added to $T|_X$ by refining $T|_X$ at *v*, and (2) Given $\pi \in C(T_1,T_2,X)$, $sv(\pi )=v$ means $\exists T'$, a refinement of *T* at *v*, so that $C(T'|_X)= C(T|_X) \cup \{\pi \}$.

*Formal proofs* We start the formal theory and proofs with a natural relationship between edges in restricted trees and restricted bipartitions.

#### **Lemma 6**

*Let* $T \in {\mathcal {T}}_S$ *and let* $\pi = [A|B] \in C(T)$ *be a bipartition induced by* $e \in E(T)$* . Let* $R \subseteq S$.

1. If $R \cap A = \emptyset$ or $R \cap B = \emptyset$, then $e \notin P(e')$ for any $e'\in E(T|_R)$.
2. If $R \cap A \ne \emptyset$ and $R \cap B \ne \emptyset$, then for any $\pi ' \in C(T|_R)$ induced by $e' \in E(T|_R)$, $\pi |_R = \pi '$ if and only if $e \in P(e')$.

#### *Proof*

Let $T_R$ be the minimal subtree of *T* that spans *R*. It follows that the leaf set of $T_R$ is *R* and $T|_R$ is obtained from $T_R$ by suppressing all degree-two vertices.

(Proof of 1) We first claim that if $R \cap A = \emptyset$ or $R \cap B = \emptyset$, then $e \notin E(T_R)$. Assume by way of contradiction that $e \in E(T_R)$. There are then two non-empty components in $T_R - e$. Since *e* induces [*A*|*B*] in *T*, the two components in $T_R - e$ have leaf sets $R \cap A$ and $R\cap B$, which contradicts the assumption that one intersection is empty. Therefore, $e \notin E(T_R)$. Furthermore, every edge $e' \in E(T|_R)$ comes from a path in $T_R$. Since $e \notin E(T_R)$, then $e \notin P(e')$ for any $e' \in E(T|_R)$.

(Proof of 2) If $R\cap A \ne \emptyset$ and $R\cap B \ne \emptyset$, then *e* is required to connect $R \cap A$ with $R \cap B$ in *T* (since *e* connects *A* with *B*). Thus, *e* is in any subtree of *T* spanning *R*; in particular, $e \in E(T_R)$. Fix any $\pi ' \in C(T|_R)$ induced by $e' \in E(T|_R)$. Note that the bipartition induced by $P(e')$ in $T_R$ equals the bipartition induced by $e'$ in $T|_R$, i.e., $\pi '$. For one direction of the proof, suppose $e \in P(e')$. Because internal vertices of $P(e')$ in $T_R$ are not adjacent to any leaves, the bipartition induced by the path $P(e')$ in $T_R$ equals the bipartition induced by any of its edges (and hence, in particular, by *e*). Since *e* induces [*A*|*B*] in *T*, it induces $[R\cap A|R\cap B]$ in $T_R$. Then $\pi ' = [R\cap A|R\cap B] = \pi |_R$. On the other hand, if $\pi |_R = \pi '$, then $\pi '$ induces $[R \cap A| R \cap B]$ in $T|_R$. It follows that $P(e')$ also induces $[R \cap A| R \cap B]$ in $T_R$. Suppose $e \in P(e^*)$ for some edge $e^*\in E(T|_R)$ such that $e^* \ne e'$. Then, by the previous argument, $\pi _{e^*} = [R\cap A|R\cap B]$, which contradicts the assumption that $e^*$ and $e'$ are different edges. Therefore, $e \in P(e')$.

$$\square $$

Lemma 7 formally states that the tree $T_{\text {init}}$ we build in line 6 of Exact-RFS-2 (Algorithm 1) maximizes the $p_2(\cdot )$ score.

#### **Lemma 7**

*For any tree* $T \in {\mathcal {T}}_S$, $p_2(T) \le |\Pi _2|$*. In particular, let* $T_{\text {init}}$ *be the tree defined in line 6 of Algorithm 1. Then,* $p_2(T_{\text {init}}) = |\Pi _2|$.

#### *Proof*

Since $T_1$ and $T_2$ have different leaf sets, $C(T_1)$ and $C(T_2)$ are disjoint. Since $\Pi _2 \subseteq C(T_1)\cup C(T_2)$, $C(T_1) \cap \Pi _2$ and $C(T_2)\cap \Pi _2$ form a disjoint decomposition of $\Pi _2$. By definition of $p_2(\cdot )$, for any tree *T* of leaf set *S*,

$$\begin{aligned} p_2(T)=&\sum _{i \in [2]} |C(T|_{S_i}) \cap C(T_i) \cap \Pi _2| \le \sum _{i\in [2]}|C(T_i) \cap \Pi _2| = |\Pi _2|. \end{aligned}$$

Fix any $\pi = [A|B] \in \Pi _2$. Suppose $\pi \in C(T_i)$ and is induced by $e \in E(T_i)$ for some $i \in [2]$. By definition of $\Pi _2$, either $A \cap X = \emptyset$ or $B \cap X = \emptyset$. By Lemma 6, $e \notin P(e')$ for any backbone edge $e' \in E(T_i|_X)$. Therefore, either *e* is an internal edge in an extra subtree in $\hbox {Extra}(T_i)$, or *e* connects one extra subtree in $\hbox {Extra}(T_i)$ to the backbone tree. In either case, the construction of $T_{\text {init}}$ ensures that *e* is also present in $T_{\text {init}}|_{S_i}$ and thus $\pi \in C(T_{\text {init}}|_{S_i})$. Therefore, each bipartition $\pi \in \Pi _2$ contributes 1 to $|C(T_{\text {init}}|_{S_i}) \cap C(T_i) \cap \Pi _2|$ for exactly one index $i \in [2]$ and thus it contributes 1 to $p_2(T_{\text {init}})$. Hence, $p_2(T_{\text {init}}) = |\Pi _2|$. $\square$

We define the function $w^*: 2^X \rightarrow {\mathbb {N}}_{\ge 0}$ as follows:

$$\begin{aligned} w^*(\pi ) = \left\{ \begin{array}{ll} 0 &{} \text{ if } \pi \not \in C(T_1,T_2,X), \\ w(e_1(\pi )) &{}\text{ if } \pi \in C(T_1|_X) \setminus C(T_2|_X), \\ w(e_2(\pi )) &{}\text{ if } \pi \in C(T_2|_X) \setminus C(T_1|_X), \\ \sum _{i \in [2]}w(e_i(\pi )) &{} \text{ else. } \end{array} \right. \end{aligned}$$

For any set *F* of bipartitions, we let $w^*(F) = \sum _{\pi \in F} w^*(\pi )$.

Lemma 8 shows that $w^*(\pi )$ represents the maximum potential increase in $p_1(\cdot )$ as a result of adding bipartition $\pi$ to $T|_X$. The proof of Lemma 8 follows the idea that for any bipartition $\pi$ of *X*, there are at most $w^*(\pi )$ edges in either $T_1$ or $T_2$ whose induced bipartitions become $\pi$ when restricted to *X*. Therefore, by only adding $\pi$ to $T|_X$, at most $w^*(\pi )$ more bipartitions get included in $C(T|_{S_1})$ or $C(T|_{S_2})$ so that they contribute to the increase of $p_1(T)$.

#### **Lemma 8**

Let $\pi = [A|B]$ be a bipartition of *X* where $X \subseteq S$. Let $T \in {\mathcal {T}}_S$ be any tree with leaf set *S* such that $\pi \notin C(T|_X)$ but $\pi$ is compatible with $C(T|_X)$. Let $T'$ be a refinement of *T* such that for all $\pi ' \in C(T'|_{S_i}) \backslash C(T|_{S_i})$ for some $i \in [2]$, $\pi '|_X = \pi$. Then, $p_1(T') - p_1(T) \le w^*(\pi )$.

#### *Proof*

By definition of $p_1(\cdot )$,

$$\begin{aligned} p_1(T') - p_1(T) =&\sum _{i \in [2]} |C(T'|_{S_i}) \cap C(T_i) \cap \Pi _1| - \sum _{i \in [2]} |C(T|_{S_i}) \cap C(T_i) \cap \Pi _1| \\ =&\sum _{i \in [2]}|(C(T'|_{S_i})\backslash C(T|_{S_i})) \cap C(T_i) \cap \Pi _1|. \end{aligned}$$

Therefore, we only need to prove that

$$\begin{aligned} \sum _{i \in [2]}|(C(T'|_{S_i})\backslash C(T|_{S_i})) \cap C(T_i) \cap \Pi _1| \le w^*(\pi ). \end{aligned}$$

We perform a case analysis, as follows: Case (1): $\pi \notin C(T_1,T_2,X)$, Case (2): $\pi \in C(T_1|_X) \Delta C(T_2|_X)$, and Case (3): $\pi \in C(T_1|_X) \cap C(T_2|_X)$.

Case 1): Let $\pi \notin C(T_1, T_2, X)$. We recall that $w^*(\pi ) = 0$. Assume for contradiction that there exists a bipartition $\pi '\in (C(T'|_{S_i})\backslash C(T|_{S_i})) \cap C(T_i) \cap \Pi _1$ for some $i \in [2]$. Since $\pi \notin C(T_1, T_2, X)$ and $\pi '|_X = \pi$, $\pi ' \notin C(T_i)$ for any $i \in [2]$. This contradicts the fact that $\pi ' \in C(T_i)$ for some $i \in [2]$. Therefore, the assumption that there exists such a bipartition $\pi '$ is wrong and $\sum _{i \in [2]}|(C(T'|_{S_i})\backslash C(T|_{S_i})) \cap C(T_i) \cap \Pi _1| = 0 \le w^*(\pi )$.

Case 2): Let $\pi \in C(T_1|_X) \Delta C(T_2|_X)$. We can assume without loss of generality that $\pi \in C(T_1|_X) \backslash$ $C(T_2|_X)$ since the other possibility is symmetrical. Then, we have $w^*(\pi ) = w(e_1(\pi ))$. Let $\pi '\in (C(T'|_{S_i})\backslash C(T|_{S_i})) \cap C(T_i) \cap \Pi _1$ for some $i \in [2]$. Then we have $\pi '|_X = \pi$ by assumption of the lemma. Since $\pi \notin C(T_2|_X)$, by Lemma 6, we have $\pi ' \notin C(T_2)$ and thus $\pi ' \in C(T_1)$. By Lemma 6, the edge that induces $\pi '$ in $T_1$ is an edge on $P(e_1(\pi ))$. Since there are $w(e_1(\pi ))$ edges on $P(e_1(\pi ))$, there are at most $w(e_1(\pi ))$ distinct bipartitions $\pi '$, proving the claim.

Case 3): Let $\pi \in C(T_1|_X) \cap C(T_2|_X)$. Then we have $w^*(\pi ) = w(e_1(\pi ))+w(e_2(\pi ))$. Fix any $\pi ' \in (C(T'|_{S_i})\backslash C(T|_{S_i})) \cap C(T_i) \cap \Pi _1$ for any $i \in [2]$. Since $\pi ' \in C(T_i)$ and $\pi '|_X = \pi \in C(T_i|_X)$, by Lemma 6, the edge $e'$ that induces $\pi '$ is an edge on $P(e_i(\pi ))$. Since there are $w(e_i(\pi ))$ edges on $P(e_i(\pi ))$, there are at most $w(e_i(\pi ))$ distinct bipartitions $\pi '$ in $(C(T'|_{S_i})\backslash C(T|_{S_i})) \cap C(T_i) \cap \Pi _1$. Therefore, for any $i \in [2]$,

$$\begin{aligned} |(C(T'|_{S_i})\backslash C(T|_{S_i})) \cap C(T_i) \cap \Pi _1| \le w(e_i(\pi )). \end{aligned}$$

Taking sum of the inequalities over $i \in [2]$, we have

$$\begin{aligned} \sum _{i \in [2]}|(C(T'|_{S_i})\backslash C(T|_{S_i})) \cap C(T_i) \cap \Pi _1| \le w(e_1(\pi )) + w(e_2(\pi )) =w^*(\pi ). \end{aligned}$$

$$\square $$

#### **Lemma 9**

For any compatible set *F* of bipartitions on *X* where $X \subseteq S$, let $T \in {\mathcal {T}}_S$ be any tree with leaf set *S* such that $C(T|_X) = F$. Then $p_1(T) \le w^*(F)$.

The proof of Lemma 9 is straightforward, and uses Lemma 8 repeatedly by adding the compatible bipartitions to the tree in any selected order.

#### **Proposition 1**

Let ${\tilde{T}}$ be the tree constructed after line 11 of Algorithm 1, then $p_1({\tilde{T}}) = w^*(\text {Triv})$.

The proof naturally follows by construction (Line 8 of Algorithm 1), and implies that the algorithm adds the trivial bipartitions of *X* (which are all in *I*) to $T|_X$ so that $p_1(T)$ reaches the full potential of adding those trivial bipartitions.

Lemma 10 will show that the auxiliary data structures of Algorithm 1 and 2 are maintaining the desired information and that the algorithm can split the vertex and perform the detaching and reattaching of the extra subtrees correctly. These invariants are important to the proof of Lemma 11.

#### **Lemma 10**

At any stage of the Algorithm 1 after line 12, we have the following invariants of *T* and the auxiliary data structures *H* and *sv*:

1. For any bipartition $\pi \in \text {NonTriv}$, $sv(\pi )$ is the vertex to split to add $\pi$ to $C(T|_X)$. For any internal vertex *v*, the set of bipartitions $H(v) \subseteq \text {NonTriv}$ is the set of bipartitions which can be added to $C(T|_X)$ by splitting *v*.
2. For any $\pi = [A|B] \in H(v)$, for all $t \in \mathcal {TR}^*(\pi )$, the root of *t* is a neighbor of *v*.
3. For any $\pi = [A|B] \in C(T|_X)$ induced by edge *e*, let *Comp*(*A*) and *Comp*(*B*) be the components containing the leaves of *A* and *B* in $T|_X - e$, respectively. Then,
  - all $t \in \mathcal {TRS}(A)$ are attached to an edge or a vertex in *Comp*(*A*)
  - all $t \in \mathcal {TRS}(B)$ are attached to an edge or a vertex in *Comp*(*B*).

#### *Proof*

We prove the invariants by induction on the number of refinement steps *k* performed on *T*. When $k=0$, we have $T = {\tilde{T}}$ and $T|_X$ is a star with leaf set *X* and center vertex ${\hat{v}}$. Thus all bipartitions in $\text {NonTriv}$ are compatible with $C(T|_X)$. For any $\pi \in \text {NonTriv}$, the center vertex ${\hat{v}}$ is the vertex to refine in $T|_X$ in order to add $\pi$ to $C(T|_X)$. Therefore, it is correct that $sv(\pi ) = {\hat{v}}$ for every $\pi \in \text {NonTriv}$ and $H({\hat{v}}) = \text {NonTriv}$. The roots of all extra subtrees in $\mathcal {TR}^*(\pi )$ for any $\pi \in \text {NonTriv}$ are all neighbors of ${\hat{v}}$, so invariant 2 also holds. We note that at this point, $C(T|_X) = \text {Triv}$. For any trivial bipartition $\pi \in C(T|_X)$, let $\pi = [\{a\}|B]$. It is easy to see that since *a* is a leaf, $\mathcal {TRS}_i(\{a\}) = \emptyset$ and $\mathcal {TRS}_i(B) = \hbox {Extra}(T_i) \backslash \mathcal {TR}(e_i(\pi ))$ for both $i \in [2]$. Then $\mathcal {TRS}(\{a\}) = \emptyset$ and $\mathcal {TRS}(B) = (\hbox {Extra}(T_1) \cup \hbox {Extra}(T_2)) \backslash \mathcal {TR}^*(\pi )$. Therefore, invariant 3(a) trivially holds as $\mathcal {TRS}(\{a\}) = \emptyset$.

Since $Comp(\{a\})$ is the vertex *a*, *Comp*(*B*) is the rest of the star of $T|_X$ excluding *a* and the edge $(a, {\tilde{v}})$ between *a* and center vertex ${\tilde{v}}$. Since $\mathcal {TRS}(B)$ does not include $\mathcal {TR}^*(\pi )$ (which are the only extra subtrees attached to edge $(a,{\tilde{v}})$), all extra subtrees in $\mathcal {TRS}(B)$ are attached to an edge or a vertex in *Comp*(*B*), and so invariant 3(b) holds. This proves invariant 3 and thus concludes our proof for the base case

Assume that all invariants hold after any $k' < k$ steps of refinement. Let $\pi = [A|B]$ be the bipartition to add in the *k*th refinement step. We will show that after the *k*th refinement step, i.e., one execution of Algorithm 2, the invariants still hold for the resulting tree $T'$. Since $v = sv(\pi )$ at the beginning of Algorithm 2, $\pi$ can be added to $C(T|_X)$ by splitting *v*. We can divide the set of neighbors of *v* in $T|_X$ into $N_A \cup N_B$ such that $N_A$ (or $N_B$ respectively) consists of neighbors of *v* that can reach vertices of *A* (or *B*) but not *B* (or *A*) in $T|_X-v$. Then, the algorithm correctly finds $N_A$ and $N_B$ and connects $N_A$ to $v_a$ and $N_B$ to $v_b$ so the new edge $(v_a,v_b)$ induces the bipartition $\pi = [A|B]$ in $T|_X$. For any vertex *u* other than *v* and any bipartition $\pi ' \in H(u)$, the invariants 1 and 2 still hold after Algorithm 2 as we do not change *H*(*u*), $sv(\pi ')$, or the extra subtrees attached to *u*. For any bipartition $\pi ' \in H(v)$ such that $\pi ' \ne \pi$, if $\pi '$ is not compatible with $\pi$, then it cannot be added to $C(T'|_X)$ since $\pi$ is added, so the algorithm correctly discards $\pi '$ and does not add it to $H(v_a)$ or $H(v_b)$. If $\pi '$ is compatible with $\pi$, we will show that the invariants 1 and 2 still hold for $\pi '$.

Fix any $\pi ' = [A'|B'] \in H(v)$ s.t. $\pi '\ne \pi$ and $\pi '$ is compatible with $\pi$. One of $A'$ and $B'$ must be a subset of one side of [*A*|*B*]. Assume without loss of generality that $A' \subseteq A$ (other cases are symmetric), so that $B \subseteq B'$. In this case, Algorithm 2 adds $\pi '$ to $H(v_a)$ and sets $sv(\pi ) = v_a$. We will show that this step preserves the invariants. Since $\pi ' \in H(v)$, before adding $\pi$ we also could have split *v* to add $\pi '$ to $C(T|_X)$. Then there exists a division of neighbors of *v* in $T|_X$ into $N_{A'}$ and $N_{B'}$ such that $N_{A'}$ (or $N_{B'}$, respectively) consists of neighbors of *v* that can reach vertices of $A'$ (or $B'$) in $T|_X - v$. It is easy to see that $N_{A'} \subseteq N_A$ and $N_B \subseteq N_{B'}$. Since $N_A \cup N_B = N_{A'} \cup N_{B'} = N_{T|_X}(v)$, we have $N_A \backslash N_{A'} = N_{B'} \backslash N_B$. Since the algorithm connects all vertices in $N_B$ are to $v_b$ in $T'$ while vertices in $N_{B'} \backslash N_B$ are connected to $v_a$, $N_{B'} \backslash N_B \cup \{v_b\}$ is the set of all neighbors of $v_a$ that can reach leaves of $B'$ in $T'|_X - v_a$. Then $N_{T'|_X}(v_a) = N_A \cup \{v_b\} = N_{A'} \cup (N_A \backslash N_{A'} \cup \{v_b\}) = N_{A'} \cup (N_{B'} \backslash N_B \cup v_b)$ implies that $N_{A'}$ and $N_{B'} \backslash N_B \cup \{v_b\}$ gives a division of neighbors of $v_a$ such that $N_{A'}$ are the neighbors that can reach leaves of $A'$ in $T'|_X -v_a$ and $N_{B'} \backslash N_B \cup \{v_b\}$ are the neighbors that can reach leaves of $B'$ in $T'|_X -v_a$. Such a division proves that $v_a$ is the correct vertex to refine in $T'|_X$ to add $\pi '$ to $C(T'|_X)$ after the *k*th refinement. Therefore, invariant 1 holds with respect to $\pi '$. Since $\pi ' \in H(v)$ before adding $\pi$, we also have for all $t \in \mathcal {TR}^*(\pi ')$, the root of *t* is a neighbor of *v* before adding $\pi$. Since $A' \subseteq A$, $\pi ' \in \mathcal {BP}(A)$ and thus $\mathcal {TR}^*(\pi ) \subseteq \mathcal {TRS}(A)$. Then, Algorithm 2 correctly attaches roots of all trees in $\mathcal {TR}^*(\pi ')$ to $v_a$. Therefore invariant 2 holds for $\pi '$.

We have shown that invariants 1 and 2 hold for the tree $T'$ with the auxiliary data structures *H* and *sv*. Next, we show that invariant 3 holds. Since $\pi$ is the only bipartition in $C(T'|_X)$ that is not in $C(T|_X)$, we only need to show two things: i) for any $\pi ' \in C(T|_X)$, the invariant 3 still holds, ii) invariant 3 holds for $\pi$. We first show i). Fix $\pi '=[A'|B'] \in C(T|_X)$. Since $\pi$ is compatible with $\pi '$, one of $A'$ and $B'$ must be a subset of one of *A* and *B*. We assume without loss of generality that $A' \subseteq A$. Therefore, $B \subseteq B'$. Let $Comp(A')$ and $Comp(B')$ be the components containing the leaves of $A'$ and $B'$ in $T|_X - e'$, where $e'$ induces $\pi '$. Since $Comp(A')$ is unchanged after the refinement, invariant 3(a) is trivially true. Since $B \subseteq B'$, *Comp*(*B*) is a subgraph of $Comp(B')$ and $v \in Comp(B')$. During the refinement, *v* is split into $v_a$ and $v_b$, both of which are still part of $Comp(B')$. Since all $t \in \mathcal {TRS}(B)$ are attached to an edge or a vertex in $Comp(B')$ before refinement and any extra subtree attached to *v* before is now on either $v_a$, or $v_b$, or $(v_a,v_b)$, all of which are part of $Comp(B')$, they are all still attached to an edge or a vertex in $Comp(B')$. Thus, the invariant 3 holds with respect to $\pi '$.

For ii), we show invariant 3(a) holds for $\pi$ and 3(b) follows the same argument. For any extra subtree in $t \in \mathcal {TRS}(A)$, if it was attached to *v* before refinement, then it is now attached to $v_a$, which is in *Comp*(*A*). If it was not attached to *v* before refinement, then let $N_B$ be as defined from Algorithm 2. For any bipartition $\pi '= [A'|B']$ induced by (*v*, *u*) where $u \in N_B$. We know that $(v,u) \in Comp(B)$ and thus either $A' \subseteq B$ or $B' \subseteq B$. Assume without loss of generality that $B'\subseteq B$. Then we have $\mathcal {BP}(B') \cup \{\pi '\} \subseteq \mathcal {BP}(B)$ and thus $\mathcal {TRS}(B') \cup \mathcal {TR}^*(\pi ') \subseteq \mathcal {TRS}(B)$. We note that $\mathcal {TRS}(A)$ and $\mathcal {TRS}(B)$ are disjoint. Since $t \in \mathcal {TRS}(A)$, we know $t \notin \mathcal {TRS}(B)$, then $t \notin \mathcal {TRS}(B')\cup \mathcal {TRS}^*(\pi ')$. Let $Comp(A')$ and $Comp(B')$ be the components containing the leaves of $A'$ and $B'$ in $T|_X - (v,u)$, respectively. Then $Comp(A')$ contains *v* and $Comp(B')$ contains *u*. Since $t \notin \mathcal {TR}^*(\pi ')$, it cannot be attached to (*v*, *u*). Also by the invariant 3 with respect to $\pi '$, *t* is not attached to any vertex or edge in $Comp(B')$. Since this is true for every neighbor of *v* in $N_B$, $t \notin Comp(B)$ as *Comp*(*B*) consists of only edges connecting *v* to a neighbor $u\in N_B$ and the component containing *u*. Since *t* was not attached to *v* before the refinement, *t* is not attached to $(v_a,v_b)$ or *Comp*(*B*) after the refinement, then *t* must be attached to some edge or vertex in *Comp*(*A*). This proves invariant 3(a) for $\pi$ and thus the inductive proof. $\square$

#### **Lemma 11**

Let *T* be a supertree computed within Algorithm 1 at line 14 immediately before a refinement step. Let $\pi = [A|B] \in \text {NonTriv}\cap I$. Let $T'$ be a refinement of *T* obtained from running Algorithm 2 with supertree *T*, bipartition $\pi$, and the auxiliary data structures *H* and *sv*. Then, $p_1(T') - p_1(T) = w^*(\pi )$.

The idea for the proof of Lemma 11 is that for any non-trivial bipartition $\pi \in I$ of *X* to be added to $T|_X$, Algorithm 2 is able to split the vertex correctly and move extra subtrees around in a way such that each bipartition in $T_1$ or $T_2$ that is induced by an edge in $P(e_1(\pi ))$ or $P(e_2(\pi ))$, which is not in $T|_{S_1}$ or $T|_{S_2}$ before the refinement, becomes present in $T|_{S_1}$ or $T|_{S_2}$ after the refinement. Since there are exactly $w^*(\pi )$ such bipartitions, they increase $p_1(\cdot )$ by $w^*(\pi )$. Now we give the formal proof.

#### *Proof*

Since *I* corresponds to an independent set in the incompatibility graph *G*, all bipartitions in *I* are compatible. Since $C(T|_X) \subseteq \text {Triv}\cup (\text {NonTriv}\cap I) = I$, $\pi \in \text {NonTriv}\cap I$ must be compatible with $C(T|_X)$, then there is a vertex to split to add $\pi$ to $C(T|_X)$. By invariant 1 of Lemma 10, $v = sv(\pi )$ is the vertex to split to add $\pi$ to $T|_X$ and thus Algorithm 2 correctly splits *v* into $v_a$ and $v_b$ and connects them to appropriate neighbors such that in $T'|_X$, $(v_a,v_b)$ induces $\pi$.

We abbreviate $e_1(\pi )$ and $e_2(\pi )$ by $e_1$ and $e_2$. We number the extra subtrees attached to $e_1$ as $t_1, t_2, \dots , t_p$, where $p = w(e_1)-1$ and $t_1$ is the closest to *A* in $T_1$. Similarly, we number the extra subtrees attached to $e_2$ as $t_1', t_2', \dots , t_q'$, where $q = w(e_2)-1$ and $t_1'$ is the closest to *A* in $T_2$. For any set ${\mathcal {T}}$ of trees, let $L({\mathcal {T}})$ denote the union of the leaf set of trees in ${\mathcal {T}}$. We note that if $e_i$ exists, $\hbox {Extra}(T_i) = \mathcal {TRS}_i(A) \cup \mathcal {TRS}_i(B) \cup \mathcal {TR}(e_i)$. Thus, $A \cup L(\mathcal {TRS}_i(A)) \cup L(\mathcal {TR}(e_i)) \cup L(\mathcal {TRS}_i(B)) \cup B = S_i$ for $i \in [2]$. For each $k \in [w(e_1)]$, we define

$$\begin{aligned} A_k := \bigcup _{i = 1}^{k-1} L(t_i) \cup L(\mathcal {TRS}_1(A)) \cup A,\; \pi _k := [A_k | S_1 \backslash A_k], \end{aligned}$$

and for each $k \in [w(e_2)]$, we define

$$\begin{aligned} A_k' := \bigcup _{i = 1}^{k-1} L(t_i') \cup L(\mathcal {TRS}_2(A)) \cup A,\; \pi _k' := [A_k' | S_2 \backslash A_k']. \end{aligned}$$

We know that for each $k \in [w(e_1)]$,

$$\begin{aligned} S_1 \backslash A_k = \bigcup _{i = k}^{p} L(t_i) \cup L(\mathcal {TRS}_1(B)) \cup B. \end{aligned}$$

Thus, for any $k \in [w(e_1)]$, $\pi _k$ is the bipartition induced by the *k*th edge on $P(e_1)$ in $T_1$, where the edges are numbered from the side of *A*. Therefore, $\pi _k \in C(T_1)$ for any $k \in [w(e_1)]$. Similarly, $\pi _k' \in C(T_2)$ for any $k \in [w(e_2)]$.

Since for any $k \in [w(e_1)]$, $A_k \cap X = A \ne \emptyset$ and $(S_1 \backslash A_k) \cap X = B \ne \emptyset$, we have $\pi _k |_X = \pi$ and $\pi _k \in \Pi _1$. Similarly, for each $k \in [w(e_2)]$, $\pi _k' \in \Pi _1$ and $\pi _k'|_X = \pi$. We also know that since $\pi \notin C(T|_X)$, by Lemma 6, $\pi _k \notin C(T|_{S_1})$ for any $k \in [w(e_1)]$ and $\pi _k' \notin C(T|_{S_2})$ for any $k \in [w(e_2)]$. We claim that $\pi _k \in C(T'|_{S_1})$ for all $k \in [w(e_1)]$ and $\pi _k' \in C(T'|_{S_2})$ for all $k \in [w(e_2)]$. Then assuming the claim is true, we have $|C(T'|_{S_1})\cap C(T_1) \cap \Pi _1| - |C(T|_{S_1})\cap C(T_1) \cap \Pi _1| = w(e_1)$ and $|C(T'|_{S_2}) \cap C(T_2) \cap \Pi _1| - |C(T|_{S_2}) \cap C(T_2) \cap \Pi _1| = w(e_2)$, and thus $p_1(T') - p_1(T) = w(e_1) + w(e_2) = w^*(\pi )$.

Now we only need to prove the claim. Fix $k \in [w(e_1)]$, we will show that $\pi _k \in C(T'|_{S_1})$. The claim of $\pi _k' \in C(T'|_{S_2})$ for any $k \in [w(e_2)]$ follows by symmetry. By invariant 2 of Lemma 10, we know that all extra subtrees of $\mathcal {TR}(e_1)$ were attached to *v* at the beginning of Algorithm 2 and thus the algorithm attaches them all onto $(v_a, v_b)$ in the order of $t_1,t_2,\dots ,t_p$, such that $t_1$ is closest to *A*. Let the attaching vertex of $t_i$ onto $(v_a, v_b)$ be $u_i$ for any $i \in [w(e_1)]$. Then we note $P( (v_a,v_b) )$ is the path from $v_a$ to $u_1,u_2,\dots , u_p$ and then to $v_b$. For any $t \in \mathcal {TRS}_1(A)$, by invariant 3 of Lemma 10, *t* is attached to *Comp*(*A*), the component containing *A* in $T'|_X - (v_a,v_b)$. Therefore, if we delete any edge of $P((v_a,v_b))$ from $T'$, *t* is in the same component as *A*. Similarly, for any $t \in \mathcal {TRS}_1(B)$, *t* is in the same component as *B* if we delete any edge of $P((v_a,v_b))$ from *T*. In particular, consider $T'|_{S_1} - (u_{k-1}, u_k)$. The component containing $u_{k-1}$ and *A* contains all of $\mathcal {TRS}_1(A)$ and $\{t_i\mid i \in [k-1]\}$, thus the leafset of that component is

$$\begin{aligned}A \cup L(\mathcal {TRS}_1(A)) \cup \bigcup _{i = 1}^{k-1}L(t_i) = A_k.\end{aligned}$$

Therefore, the edge $(u_{k-1},u_k)$ induces the bipartition $[A_k | S_1 \backslash A_k]$ in $T'|_{S_1}$. Hence, $\pi _k \in C(T'|_{S_1})$ as desired. $\square$

#### **Proposition 2**

Let *G* be the weighted incompatibility graph on $T_1|_X$ and $T_2|_X$, and let *I* be the set of bipartitions associated with vertices in $I^*$, which is a maximum weight independent set of *G*. Let *F* be any compatible subset of $C(T_1,T_2,X)$. Then $w^*(I) \ge w^*(F)$.

#### *Proof*

We extend the weight function of vertices in *G* and let $\text {weight}(U)$ denote the total weight of any set *U* of vertices of *G*. For any compatible subset of bipartitions $F \subseteq C(T_1,T_2,X)$, let *V*(*F*) be the set of vertices of *G* associated with the bipartitions in *F*. We first claim that $w^*(F) = \text {weight}(V(F))$. For each $\pi \in C(T_1|_X) \cap C(T_2|_X)$, there are two vertices associated with it in *G* with a total weight of $w(e_1(\pi ))+w(e_2(\pi ))$, which is exactly $w^*(\pi )$. For each $\pi \in C(T_i|_X) \backslash C(T_j|_X)$ for $i,j \in [2]$ and $i \ne j$, let $v_\pi$ be the only vertex for $\pi$ in *G*, then $\text {weight}(v_\pi ) = w(e_i(\pi )) = w^*(\pi )$. Since $C(T_1,T_2,X) = (C(T_1|_X) \cap C(T_2|_X) \cup (C(T_1|_X) \Delta C(T_2|_X))$, we have shown that the $w^*(\pi ) = \text {weight}(V(\{\pi \}))$ for any $\pi \in C(T_1,T_2,X)$. Then taking the sum over all $\pi \in F$, we have $w^*(F) = \text {weight}(V(F))$. Since $V(I) = I^*$, we have $w^*(I) = \text {weight}(V(I)) = \text {weight}(I^*)$. Fix any compatible subset *F* of $C(T_1,T_2,X)$. Let $F' = F \backslash (C(T_1|_X) \cap C(T_2|_X))$. Then we have

$$\begin{aligned} w^*(F) =&w^*(F') + w^*(F \cap C(T_1|_X) \cap C(T_2|_X)) \\ \le&w^*(F') + w^*(C(T_1|_X) \cap C(T_2|_X)) \\ =&w^*(F' \cup (C(T_1|_X) \cap C(T_2|_X))) \\ =&\text {weight}(V(F' \cup (C(T_1|_X) \cap C(T_2|_X)))). \end{aligned}$$

Since *F* is compatible, $F' \cup (C(T_1|_X) \cap C(T_2|_X))$ is also compatible, and thus $V(F' \cup (C(T_1|_X) \cap C(T_2|_X)))$ is an independent set in *G*. Therefore, $\text {weight}(V(F' \cup (C(T_1|_X) \cap C(T_2|_X)))) \le \text {weight}(I^*)$, since $I^*$ is a maximum weight independent set in *G*. We conclude that $w^*(F) \le \text {weight}(V(F' \cup (C(T_1|_X) \cap C(T_2|_X)))) \le \text {weight}(I^*) = w^*(I)$. $\square$

We now restate and prove Theorem 1:

**Theorem 1** *Let* ${\mathcal {A}} = \{T_1,T_2\}$ *with* $S_i$ *the leaf set of* $T_i$ ($i=1,2$) *and* $X:= S_1 \cap S_2$. *The problems* $\textsc {RFS-2}$-$\textsc {B}$(${\mathcal {A}}$) *and* $\textsc {SFS-2}$-$\textsc {B}$(${\mathcal {A}}$) *can be solved in* $O(n^2|X|)$ *time, where* $n :=\max \{|S_1|,|S_2|\}$.

#### *Proof*

First we claim that $p_1(T^*) \ge p_1(T)$ for any tree $T \in {\mathcal {T}}_S$, where $T^*$ is defined as from line 14 of Algorithm 1. Fix arbitrary $T \in {\mathcal {T}}_S$ and let $F = C(T|_X)$. Then by Lemma 9, $p_1(T) \le w^*(F)$. We know that $w^*(\pi ) = 0$ for any $\pi \notin C(T_1, T_2, X)$, so $w^*(F) = w^*(F \cap C(T_1, T_2, X))$ and thus $p_1(T) \le w^*(F \cap C(T_1, T_2, X))$. Since $F \cap C(T_1, T_2, X)$ is a compatible subset of $C(T_1, T_2, X)$, we have $w^*(F \cap C(T_1, T_2, X)) \le w^*(I)$ by Proposition 2. Then $p_1(T) \le w^*(I)$. Since $\text {Triv}\subseteq C(T_1|_X) \cap C(T_2|_X) \subseteq I$, we have $I = (\text {NonTriv}\cap I) \cup (\text {Triv}\cap I) = (\text {NonTriv}\cap I) \cup \text {Triv}.$ Therefore, by Proposition 1 and Lemma 11, we have

$$\begin{aligned} p_1(T^*)&= p_1({\tilde{T}}) + \sum _{\pi \in \text {NonTriv}\cap I}w^*(\pi ) = w^*(\text {Triv}) + w^*(\text {NonTriv}\cap I) = w^*(I). \end{aligned}$$

Therefore, $$p_1(T^*) = w^*(I) \ge p_1(T)$$.

From Lemma 7 and the fact that a refinement of a tree never decreases $p_1(\cdot )$ and $p_2(\cdot )$, we also know that $p_2(T^*) \ge p_2(T_{\text {init}}) \ge p_2(T)$ for any tree $T \in {\mathcal {T}}_S$. Since for any $T \in {\mathcal {T}}_S$, $\text {SF}(T, {\mathcal {A}}) = p_1(T) + p_2(T)$, $T^*$ achieves the maximum split support score with respect to ${\mathcal {A}}$ among all trees in ${\mathcal {T}}_S$. Thus, $T^*$ is a solution to $\textsc {Relax}$—$\textsc {SFS-2}$-$\textsc {B}$ (Corollary 1). If $T^*$ is not binary, Algorithm 1 arbitrarily resolves every high degree node in $T^*$ until it is a binary tree and then returns a tree that achieves the maximum split support score among all binary trees of leaf set *S*.

We give the running time analysis for Algorithm 1 to complete the proof of Theorem 1. First we analyze the running time of Algorithm 2, i.e., one refinement step. Dividing the neighbors of *v* and connecting them to $v_a$ and $v_b$ appropriately in lines $3-6$ take $O(|X|^2)$ time. We can do a depth-first-search in $T|_X - v$ from every neighbor *u* of *v* and check in *O*(|*X*|) time if any newly discovered vertex is in *A* or *B* and connect *u* to $v_a$ or $v_b$ accordingly. Moving extra subtrees in $\mathcal {TR}^*(\pi )$ in line 7 takes *O*(*n*) time as $T_i$ has at most *n* leafs and thus there are *O*(*n*) extra subtrees in total, so $|\mathcal {TR}^*(\pi )|$ is *O*(*n*). Lines $8-13$ take *O*(*n*) time as the mappings are pre-calculated and there are again *O*(*n*) extra subtrees to be moved. Updating the data structures in lines $15-21$ takes $O(|X|^2)$ time as there are at most *O*(|*X*|) bipartitions in *H*(*v*) and each of the containment conditions is checkable in *O*(|*X*|) time by checking whether one side of $\pi '$ is a subset of one side of $\pi$ (assuming that labels of leaves in both sides of the bipartitions are stored as pre-processed sorted lists instead of sets). The rest of the algorithm takes constant time. Overall, Algorithm 2 runs in $O(n + |X|^2)$ time.

Next we analyze the running time for Algorithm 1, i.e., Exact-RFS-2. Computing $C(T_1|_X)$ and $C(T_2|_X)$ in line 1 takes $O(n^2+ n|X|^2)$ time as we need to compute $\pi _e|_X$ for all $e \in E(T_1) \cup E(T_2)$ and then take the union. There are *O*(*n*) edges in $E(T_1) \cup E(T_2)$. Computing $\pi _e|_X$ for each edge takes *O*(*n*) time by running DFS on $T_i - e$ to obtain $\pi _e$ and then taking intersection of both sides of $\pi _e$ with *X*, separately. Together it takes $O(n^2)$ time. Taking union of the bipartitions takes $O(n |X|^2)$ time as there are *O*(*n*) bipartitions to add and whenever we add a new bipartition, it needs to be compared to the *O*(|*X*|) distinct existing ones in the set. Since all bipartitions have size *O*(|*X*|), the comparison can be done in *O*(|*X*|) time (if each of them is represented by two sorted lists instead of two sets). In this step, we can always maintain a set of edges in $T_i$ for each bipartition $\pi \in C(T_1,T_2,X)$ such that $\pi _e|_X = \pi$.

Lines $2-5$ compute the mappings and values we need in latter part of the algorithm. We analyze the running time for each $\pi = [A|B] \in C(T_1,T_2,X)$ first. We can compute the path $P(e_i(\pi ))$ by assembling the set of edges associated with $\pi$ in $T_i$ from the last step into a path. This takes $O(n^2)$ time by counting the times any vertex appear as an end vertex in the set of edges. The two vertices appearing once are the end vertices of the path while those appearing twice are internal vertices of the path. Then $w(e_i(\pi )) = |P(e_i(\pi ))|$ can be found in constant time. Then we can find $\mathcal {TR}(e_i(\pi ))$ by DFS in $T_i - v$ for every internal node *v* of $P(e_i(\pi ))$, starting the search from the unique neighbor *u* of *v* such that *u* does not appear in the path. This takes *O*(*n*) time. We compute $\mathcal {BP}_i(A)$ and $\mathcal {BP}_i(B)$ by iterating over *O*(|*X*|) bipartitions in $C(T_i|_X)$ and check if one side of any bipartition is a subset of *A* or *B* in *O*(|*X*|) time, this takes $O(|X|^2)$ time together. Next, we compute $\mathcal {TRS}_i(A)$ (or $\mathcal {TRS}_i(B)$) by taking unions of extra subtrees in $\mathcal {TR}(e_i(\pi ))$ for any $\pi \in \mathcal {BP}_i(A)$ (or $\mathcal {BP}_i(B)$) in *O*(*n*) time. Extra subtrees are uniquely identified by their roots and $\mathcal {TR}(e_i(\pi ))$ is disjoint from the set of extra subtrees associated with other edges, so taking union of at most *O*(*n*) extra subtrees takes *O*(*n*) time. Therefore, all the mappings and values can be computed in $O(n^2)$ time for each bipartition and thus it takes $O(n^2|X|)$ time overall. With all the extra subtrees calculated for each partition, we can compute $\hbox {Extra}(T_i)$ in $O(n^2)$ time.

Constructing $T_{\text {init}}$ in line 6 takes *O*(*n*) time. Line 7 constructs an incompatibility graph with *O*(|*X*|) vertices and $O(|X|^2)$ edges in $O(|X|^3)$ time as compatibility of any pair of bipartitions of size *O*(|*X*|) can be checked in *O*(|*X*|) time. For line 8, we can reduce Maximum Weight Independent Set to Minimum Cut problem in a directed graph with a dummy source and sink. Then the Minimum Cut problem can be solved by a standard Maximum Flow Algorithm. Since the best Maximum Flow algorithm runs in *O*(|*V*||*E*|) time and the graph has *O*(|*X*|) vertices and $O(|X|^2)$ edges, this line runs in $O(|X|^3)$ time. Line 10-11 essentially runs line 7 of Algorithm 2 *O*(|*X*|) times using a total of *O*(*n*|*X*|) time. Line 12 initiates the data structure *H* and *sv* in *O*(|*X*|) time. Lines $13-14$ run Algorithm 2 *O*(|*X*|) times with a total of $O(n|X|+|X|^3)$ time. Since $|X| \le n$, $|X|^3 \le n|X|^2 \le n^2|X|$, and thus, the overall running time of the algorithm is dominated by the running time of lines $2-5$, which is $O(n^2|X|)$.

$$\square $$

#### **Corollary 1**

Let ${\mathcal {A}} = \{T_1,T_2\}$ with $S_i$ the leaf set of $T_i$ ($i=1,2$) and $X:= S_1 \cap S_2$. $\textsc {Relax}$—$\textsc {SFS-2}$-$\textsc {B}$ can be solved in $O(n^2|X|)$ time, where $n :=\max \{|S_1|,|S_2|\}$.

### Using Exact-RFS-2 in Divide-and-Conquer methods

Here we present a divide-and-conquer method for phylogeny estimation, which uses the Exact-RFS-2 algorithm in combination with DACTAL [11], a prior divide-and-conquer framework.

Let $\Phi$ be a model of evolution (e.g., GTR) for which statistically consistent methods exist, and we have some data (e.g., sequences) generated by the model and wish to construct a tree. We construct an initial estimate of the tree, and we select an edge *e* in the tree. The deletion of *e* and its endpoints creates four subtrees, and we let *P* be the set of the *p* nearest leaves to *e* taken from each subtree (including all leaves that tie for closest in each subtree). We define the subsets be $A \cup P$ and $B \cup P$, where $\pi _e = [A|B]$), and we re-estimate trees on these subsets and then combine the trees together using Exact-RFS-2. We call this the DACTAL-Exact-RFS-2 pipeline, due to its similarity to the DACTAL pipeline [11]. The DACTAL pipeline differs from the DACTAL-Exact-RFS-2 pipeline only in that it computes four trees (each containing the set *P* and otherwise being leaf-disjoint) and then combines the overlapping subset trees using the Strict Consensus Merger technique, and was proven statistically consistent when the subset trees are computed using statistically consistent methods.

Before we prove that DACTAL-Exact-RFS-2 can enable statistically consistent pipelines, we begin with some definitions. Given a tree *T* and an internal edge *e* in *T*, the deletion of the edge *e* and its endpoints defines four subtrees. A **short quartet around** *e* is a set of four leaves, one from each subtree, selected to be closest to the edge. Note that due to ties, there can be multiple short quartets around some edges. The set of short quartets for a tree *T* is the set of all short quartets around the edges of *T*. The **short quartet trees of** *T* is the set of quartet trees on short quartets induced by *T*. It is well known that the short quartet trees of a tree *T* define *T*, and furthermore *T* can be computed from this set in polynomial time [33–35].

#### **Lemma 12**

Let *T* be a binary tree on leaf set *S* and let $$A,B \subseteq S$$. Let $$T_A=T|_A$$ and $$T_B=T|_B$$ (i.e., $$T_A$$ and $$T_B$$ are induced subtrees). If every short quartet tree is induced in $$T_A$$ or in $$T_B$$, then *T* is the unique compatibility supertree for $$T_A$$ and $$T_B$$ and Exact-2-RFS($$T_A,T_B) = T$$.

#### *Proof*

Because $T_A$ and $T_B$ are induced subtrees of *T*, it follows that *T* is a compatibility supertree for $T_A$ and $T_B$. Furthermore, because every short quartet tree appears in at least one of these trees, *T* is the unique compatibility supertree for $T_A$ and $T_B$ (by results from [34, 35], mentioned above). Finally, because *T* is a compatibility supertree, the RFS score of *T* with respect to $T_A,T_B$ is 0, which is the best possible. Since Exact-2-RFS solves the RFS problem on two binary trees, Exact-2-RFS returns *T* given input $T_A$ and $T_B$. $\square$

Thus, Exact-2-RFS is guaranteed to return the true tree when given two correct trees that have sufficient overlap (in that all short quartets are included). We continue with proving that these pipelines are statistically consistent.

#### **Theorem 2**

The DACTAL-Exact-RFS-2 pipeline is a statistically consistent method for estimating the unrooted tree topology under any model $\Phi$ for which statistically consistent unrooted tree topology estimation methods exist.

#### *Proof*

The proof is very similar to the proof given for the original DACTAL pipeline in [11]. Let $$\Phi $$ be the stochastic evolution model. To establish statistical consistency of the DACTAL-Exact-RFS-2 pipeline (see above), we need to prove that as the amount of data increases the unrooted tree topology that is returned by the pipeline converge to the true unrooted tree topology. That is, we will show that for any $$\epsilon >0$$, there is an amount of data so that the probability of returning the true tree topology given that amount of data is at least $$1-\epsilon $$. Hence, let *F* be the method used to compute the starting tree, let *G* be the method used to compute the subset trees, and let $$\epsilon >0$$ be given. Because *F* is statistically consistent under $$\Phi $$, it follows that there is an amount of data so that the starting tree computed by *F* will have the true tree topology *T* with probability at least $$1-\epsilon /2$$. Now consider the decomposition into two sets produced by the algorithm produced by deleting edge *e*, applied to a tree with the true unrooted tree topology. Note that for any $$p \ge 1$$, all the leaves appearing in any short quartet around *e* are placed in the set *P*. Now, subset trees are computed using *G* on $$A \cup P$$ and $$B\cup P$$, where $$\pi _e = [A|B]$$, which we will refer to as $$T_A$$ and $$T_B$$, respectively. Since *G* is statistically consistent, for a large enough amount of data, $$T_A $$ and $$T_B$$ will have the true tree topology on their leaf sets ($$T|_{L(T_A)}$$ and $$T|_{L(T_B)}$$, respectively) with probability at least $$1-\epsilon /2$$. When $$T_A$$ and $$T_B$$ are equal to the true trees on their leaf sets, then every short quartet tree of *T* is in $$T_A$$ or $$T_B$$, so that by Lemma 12, *T* is the only compatibility supertree for $$T_A$$ and $$T_B$$. Thus, under these conditions, Exact-2-RFS($$T_A,T_B$$) returns *T*. Hence, for a large enough amount of data, Exact-2-RFS($$T_A,T_B$$) returns *T* with probability at least $$1-\epsilon $$, completing our proof. $$\square $$

Hence, DACTAL+Exact-2-RFS is statistically consistent under all standard molecular sequence evolution models and also under the MSC+GTR model [36, 37] where gene trees evolve within species trees under the multi-species coalescent model (which addresses gene tree discordance due to incomplete lineage sorting [38]) and then sequences evolve down each gene tree under the GTR model.

Note that all that is needed to guarantee that the pipeline is statistically consistent is that the method *F* used to compute the starting tree and the method *G* used to compute the subset trees be statistically consistent. However, for the sake of improving empirical performance, *F* should be fast so that it can run on the full dataset but *G* can be more freely chosen, since it will only be run on smaller datasets. Indeed, the user can specify the size of the subsets that are analyzed, with smaller datasets enabling the use of more computationally intensive methods. Thus, when estimating gene trees under the GTR [39] sequence evolution model, *F* could be a fast distance-based method, such as neighbor joining [40] and *G* could be maximum likelihood, and when estimating species trees under the MSC+GTR model, then *F* could be ASTRID [29] or ASTRAL [41] (both polynomial time methods that are statistically consistent) while *G* could be an expensive method, such as StarBeast2 [42], a Bayesian method for co-estimating gene trees and species trees under the MSC+GTR model.

When empirical performance is the main objective and statistical consistency is not a requirement, then other options are available. For example, although heuristics for maximum likelihood are not provably statistically consistent (since they are not guaranteed to find optimal solutions), they can be highly accurate in practice. Therefore, when estimating trees under the GTR [39] model, *F* could be a distance-based method, such as neighbor joining [40], and *G* could be a good maximum likelihood heuristic, such as RAxML [43] or IQ-TREE 2 [?].

## Conclusions

The main contribution of this paper is Exact-2-RFS, a polynomial time algorithm for the Robinson-Foulds Supertree (RFS) of two binary trees. We established equivalence between RFS and some other supertree problems, while also showing that solving the RFS of three or more trees is NP-hard. Finally, showed that this approach can be used in a divide-and-conquer pipeline to enable statistically consistent phylogeny estimation under sequence evolution models (e.g., GTR [39] and the hierarchical MSC+GTR model [37]).

This study advances the theoretical understanding of several established supertree problems, showing that supertree methods have the theoretical potential to be useful in phylogeny estimation on large datasets. Some prior studies (e.g., [11, 44]) have evaluated supertree methods in the context of divide-and-conquer phylogeny estimation, which is an approach championed by Wilkinson [10]. Hence, a valuable direction for future work would evaluate these and other supertree methods in order to evaluate the utility and benefits of using such approaches on biological datasets.
